# Coenzyme Q10 Improves Lipid Metabolism and Ameliorates Obesity by Regulating CaMKII-Mediated PDE4 Inhibition

**DOI:** 10.1038/s41598-017-08899-7

**Published:** 2017-08-15

**Authors:** Zhe Xu, Jia Huo, Xin Ding, Mu Yang, Lin Li, Jian Dai, Kazunori Hosoe, Hiroshi Kubo, Masayuki Mori, Keiichi Higuchi, Jinko Sawashita

**Affiliations:** 1 Department of Aging Biology, Institute of Pathogenesis and Disease Prevention, Shinshu University Graduate School of Medicine, Matsumoto, 390-8621 Japan; 2 Supplemental Nutrition Division, Pharma & Supplemental Nutrition Solutions Vehicle, Kaneka Corporation, Osaka, 530-8288 Japan; 3 Department of Advanced Medicine for Heath Promotion, Institute for Biomedical Sciences, Interdisciplinary Cluster for Cutting Edge Research, Shinshu University, Matsumoto, 390-8621 Japan; 4 Department of Biological Sciences for Intractable Neurological Diseases, Institute for Biomedical Sciences, Interdisciplinary Cluster for Cutting Edge Research, Shinshu University, Matsumoto, 390-8621 Japan

## Abstract

Our recent studies revealed that supplementation with the reduced form of coenzyme Q10 (CoQ_10_H_2_) inhibits oxidative stress and slows the process of aging in senescence-accelerated mice. CoQ_10_H_2_ inhibits adipocyte differentiation and regulates lipid metabolism. In the present study, we show that dietary supplementation with CoQ_10_H_2_ significantly reduced white adipose tissue content and improved the function of brown adipose tissue by regulating expression of lipid metabolism-related factors in KKAy mice, a model of obesity and type 2 diabetes. In the liver, CoQ_10_H_2_ reduced cytoplasmic Ca^2+^ levels and consequently inhibited the phosphorylation of CaMKII. CoQ_10_H_2_ also regulated the activity of the transcription factor C-FOS and inhibited gene expression of PDE4, a cAMP-degrading enzyme, via the CaMKII-MEK1/2-ERK1/2 signaling pathway, thereby increasing intracellular cAMP. This increased cAMP activated AMPK, enhanced oxidative decomposition of lipids, and inhibited *de novo* synthesis of fatty acids, inhibiting the development and progression of obesity and type 2 diabetes. These results suggest that CoQ_10_H_2_ supplementation may be useful as a treatment for metabolic disorders associated with obesity.

## Introduction

Imbalance between energy input and output can lead to the accumulation of excess fat, causing obesity. Obesity and metabolic disorders have become a global health problem^1^. Obesity is an important risk factor for various metabolic disorders, including insulin resistance and type 2 diabetes^2^, atherosclerosis^3^, cardiovascular disease^4^, and chronic kidney disease^5^, and can cause oxidative stress^6, 7^, endoplasmic reticulum (ER) stress^8, 9^, and mitochondrial dysfunction^10, 11^. Therefore, there is an urgent need to find a safe and effective treatment for obesity.

Increasing evidence suggests that increased oxidative stress caused by obesity is involved in the pathogenesis of metabolic syndrome^6^. Oxidative stress, caused by imbalance between the production of oxygen free radicals and the antioxidant capacity of cells, induces cell damage and abnormal production of adipocytokines, which directly leads to a series of metabolic abnormalities, including obesity-related insulin resistance, hypertension, abnormal blood lipids, and fatty degeneration of the liver^12–14^.

The ER is one of the most important intracellular signal transduction organelles^15^. It activates numerous cellular functions by releasing Ca^2+^ and inhibits them by Ca^2+^ re-uptake through skeletal sarco-endoplasmic reticulum Ca^2+^-transporting ATPase 2 (SERCA2). Recent studies showed that in obese/diabetic animal models, SERCA2 dysfunction is induced and cytoplasmic Ca^2+^ is increased, which then activates the cytoplasmic calcium-sensitive kinase, calcium/calmodulin dependent-protein kinase II (CaMKII), and triggers ER stress^16^. ER stress induced by a high fat diet and consequent metabolic disorders can be ameliorated by enhancing the function of SERCA2 and improving ER Ca^2+^ load capacity^17, 18^.

Mitochondrial dysfunction has also been shown to be associated with the development of obesity and insulin resistance^10, 11^. Peroxisome proliferator-activated receptor γ coactivator 1α (PGC-1α), a nuclear transcription coactivator, plays several roles in energy metabolism, including involvement in adaptive thermogenesis, mitochondrial biogenesis, hepatic gluconeogenesis, and β-oxidation of fatty acids^19–22^. Studies have shown that decreased PGC-1α mRNA is associated with insulin resistance^23, 24^. The Sirtuin family has become known as a key regulator of the nutrient-sensitive metabolic regulatory pathway^25^. Activation of SIRT1 promotes β-oxidation of fatty acids, prevents diet-induced nonalcoholic fatty liver disease, and reduces insulin resistance^26–28^. Obesity reduces SIRT1 activity in liver and adipose tissue^29, 30^. Increased expression of PGC-1α and SIRT1 promotes the browning of white adipose tissue and ameliorates obesity and metabolic disorders^23, 31^.

Coenzyme Q10 (CoQ_10_) is a fat-soluble micronutrient synthesized in nearly all human cells and plays a role in electron transport in the mitochondrial respiratory chain^32, 33^. CoQ_10_ content in organs is gradually decreased with age and this decline is closely associated with the occurrence and development of various diseases^34^. Therefore, intake of exogenous CoQ_10_ could help prevent the occurrence and progression of age-related diseases such as cardiovascular disease, metabolic syndrome, diabetes mellitus, cardiac dysfunction, and neurodegenerative diseases^35–38^. CoQ_10_ is enzymatically maintained in its reduced form (CoQ_10_H_2_) and also acts as a fat-soluble antioxidant to potently protect lipid membranes and lipoproteins from oxidative damage and to prevent DNA damage^39–41^. Our previous studies showed that CoQ_10_H_2_ increases cAMP and enhances the activity of SIRT1 and PGC-1α, thereby improving mitochondrial function and inhibiting oxidative stress^42^.

In addition, other studies have shown that CoQ_10_H_2_ content in adipose tissue gradually decreased with the development of obesity in both mice and humans, and that CoQ_10_H_2_ synthesis-related enzymes were upregulated as a compensatory measure^43^. CoQ_10_H_2_ also inhibits adipocyte differentiation and cholesterol synthesis^44^, but the mechanism remains unclear.

The present study shows that dietary supplementation of KKAy mice, a widely used model of diabetes and obesity, with CoQ_10_H_2_ inhibited weight gain and reduced white adipose tissue content while enhancing brown adipose tissue function, and increasing the metabolic rate. CoQ_10_H_2_ treatment also increased expression of *Sirt1*, *Pgc-1α* and *Pparα*, enhanced mitochondrial function and promoted β-oxidation of fatty acids in the liver, as well as increased levels of intracellular cAMP. We also found that CoQ_10_H_2_ enhanced the expression and function of SERCA2 and inhibited the increase of cytoplasmic Ca^2+^, and subsequently inhibited activity of the transcription factor C-FOS, which in turn inhibited the expression of phosphodiesterase 4 (PDE4) in the *in vitro* experiments. Our results demonstrate that dietary CoQ_10_H_2_ can suppress lipid accumulation and mitigate metabolic dysfunction.

## Results

### CoQ_10_H_2_ inhibited weight gain and improved metabolic syndrome in KKAy mice

**Figure 1:**
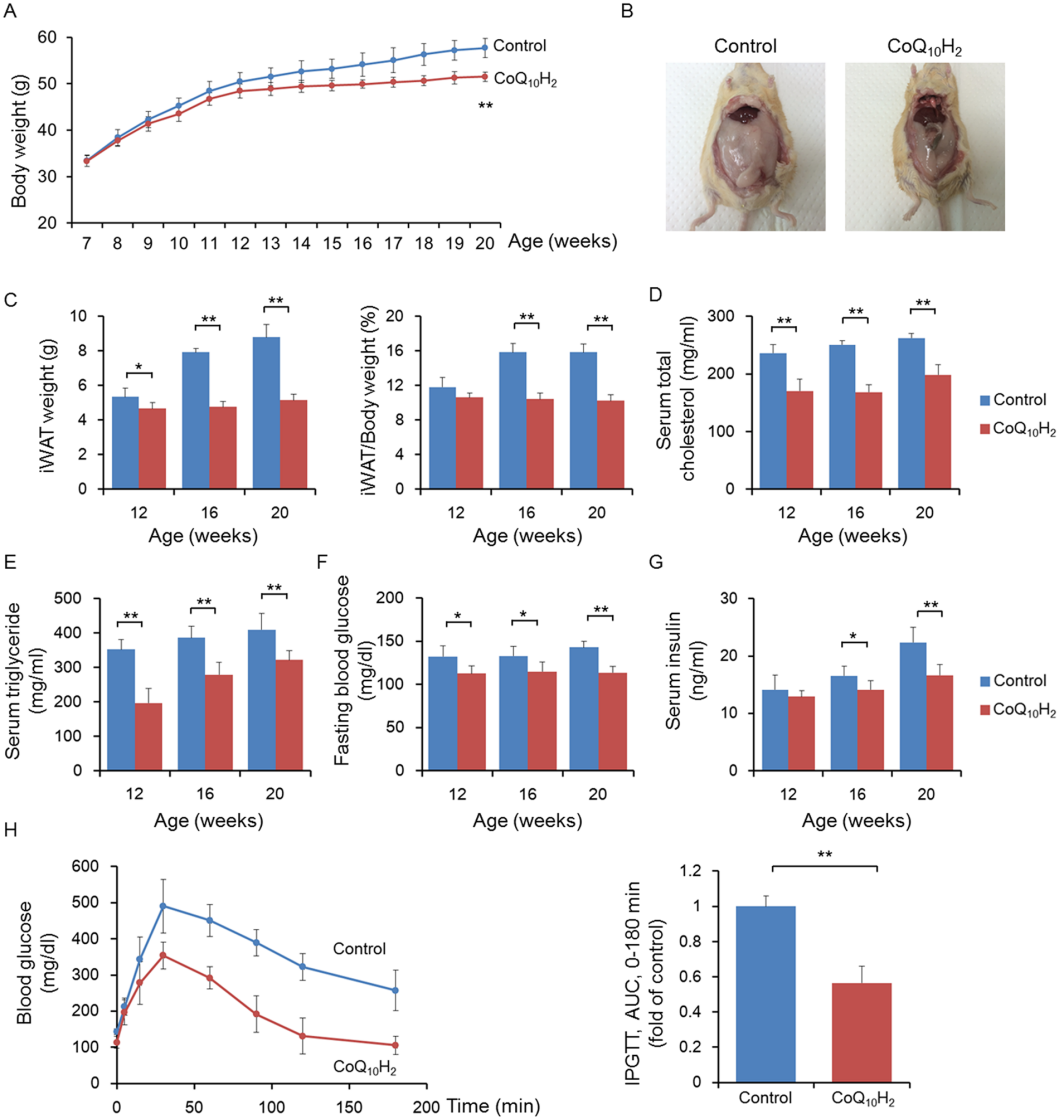
CoQ_10_H_2_ prevented the accumulation of visceral fat (iWAT) in KKAy mice and protected mice from insulin resistance and metabolic disorders. (**A**–**C**) Body weight and iWAT content changes for animals in the control and CoQ_10_H_2_ groups. Photographs show representative gross morphology of an iWAT mouse aged 20 weeks (n = 4–6, ^∗^p < 0.05, ^∗∗^p < 0.01; mean ± SD, Student’s t-test); (**D**–**G**) Serum cholesterol and triglyceride, fasting blood glucose and serum insulin levels of KKAy mice after 12 hours of fasting in each group at age 12, 16, and 20 weeks. (n = 4–6, ^∗^p < 0.05, ^∗∗^p < 0.01; mean ± SD, Student’s t-test); (**H**) Changes in blood glucose and area under the curve (AUC) in 20-week-old mice after intraperitoneal injection of glucose (1 g/kg body weight) following 12 hours of fasting. (n = 4–6, ^∗^p < 0.05, ^∗∗^p < 0.01; mean ± SD, Student’s t-test).

In this study, dietary supplementation with CoQ_10_H_2_ was employed to investigate the effect of CoQ_10_H_2_ on metabolic syndrome in KKAy mice. Every week, all mice were weighed and food intake was calculated. Compared with the control group, the body weight of KKAy mice was reduced by 12% after 12 weeks of CoQ_10_H_2_ supplementation (Fig. 1A), although the food intake was similar between the two groups at each time point (data not shown).

In addition, inguinal white adipose tissue (iWAT) was significantly reduced and percent adipose tissue was lower in KKAy mice supplemented with CoQ_10_H_2_ compared with the control group (Fig. 1B and C). The occurrence and development of obesity are often associated with abnormal lipid and glucose metabolism. Therefore, we next addressed whether CoQ_10_H_2_ can improve metabolic function. Serum total cholesterol and triglyceride content were significantly decreased in KKAy mice given CoQ_10_H_2_ supplementation (Fig. 1D and E). CoQ_10_H_2_ also reduced concentrations of fasting blood glucose and serum insulin in KKAy mice (Fig. 1F and G). In addition, mice with CoQ_10_H_2_ supplementation also showed better glucose tolerance in an intraperitoneal glucose tolerance test (IPGTT) (Fig. 1H). Together, these results indicate that CoQ_10_H_2_ can control obesity and improve insulin resistance in KKAy mice.

### CoQ_10_H_2_ prevented an increase in adipocytes in iWAT and enhanced the function of brown adipose tissue (BAT) in KKAy mice

**Figure 2:**
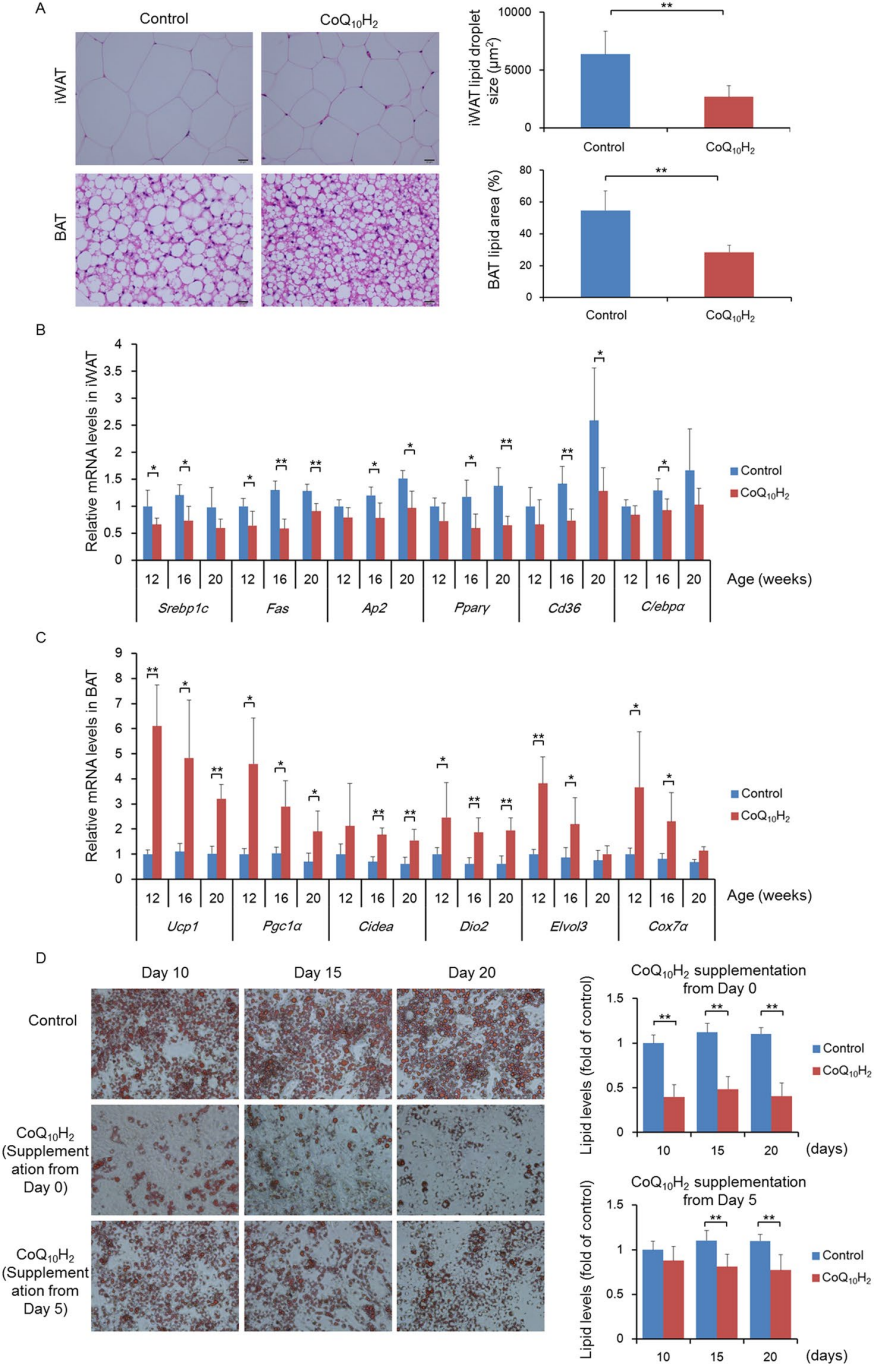
CoQ_10_H_2_ inhibited lipid accumulation in iWAT and promoted BAT function. (**A**) Images of iWAT and BAT stained with H&E in 20-week-old KKAy mice, and iWAT adipocyte size and percentage lipid content in BAT measured by Image-pro. Bar = 50 μm. (n = 4–6, ^∗∗^p < 0.01; mean ± SD, Student’s t-test); (**B**,**C**) Expression of genes involved in triglyceride and cholesterol biosynthesis and lipid mobilization related factors in iWAT and expression of genes related to mitochondrial function in BAT from mice in the control and CoQ_10_H_2_ groups at the ages of 12, 16, and 20 weeks by real-time PCR. Histograms show fold-change in mRNA level relative to 12-week-old control KKAy mice (n = 4–6, ^∗^p < 0.05, ^∗∗^p < 0.01; mean ± SD, Student’s t-test); (D) Lipid content in 3T3L1 preadipocytes after CoQ_10_H_2_ (10 μM) was added to cell cultures before (Day 0) and after (Day 5) differentiation. Quantitative analysis of lipid deposition was measured by optical density (OD) values at 510 nm after oil-red O staining. (n = 9, ^∗∗^p < 0.01; mean ± SD, Student’s t-test).

In order to demonstrate the effect of CoQ_10_H_2_ on adipocytes in iWAT from KKAy mice, iWAT from control and experimental mice was stained with hematoxylin and eosin (H&E), and expression of lipid metabolism-related factors was detected (Fig. 2A and B). Obesity in KKAy mice was primarily caused by hypertrophy of adipocytes, while CoQ_10_H_2_ supplementation significantly prevented this adipocyte hypertrophy. Expression levels of several marker genes for fatty acid synthesis (e.g., *Srebp1c* and others) and adipocyte differentiation (e.g., *Pparγ* and others) in KKAy mice iWAT showed a progressive tendency to increase with the development of obesity, whereas CoQ_10_H_2_ supplementation significantly prevented these changes, suggesting that metabolic dysfunction of adipose tissue is mitigated by dietary supplementation with CoQ_10_H_2_. BAT is involved in regulation of energy metabolism and obesity. Activation of BAT can burn fatty acids to produce heat, reduce triglyceride content, and inhibit obesity^45^. Effects of CoQ_10_H_2_ on BAT morphology were observed using H&E staining (Fig. 2A). KKAy mice in the control group had more abundant and larger lipid droplets in BAT, while CoQ_10_H_2_ supplementation inhibited the excessive accumulation of lipid droplets in BAT. BAT thermogenesis induces expression of uncoupling protein 1 (*Ucp1)* and other genes, promoting lipolysis, mitochondrial biogenesis, and β-oxidation of fatty acids. Our experiment showed that mRNA expression of *Ucp1* and other thermogenesis-related genes was significantly increased in BAT from KKAy mice supplemented with CoQ_10_H_2_ compared with the control group, suggesting that the CoQ_10_H_2_ group had increased BAT thermogenic activity (Fig. 2C).

To directly demonstrate the effect of CoQ_10_H_2_ on adipocyte differentiation and lipolysis, 10 μM CoQ_10_H_2_ was added to pre-adipocyte 3T3L1 cultures before (Day 0) and after (Day 5) differentiation, and lipid accumulation was quantitatively determined by oil red O staining (Fig. 2D). After differentiation, mature adipocytes were round and contained a large number of lipid droplets (Day 5). Compared with the control group, the addition of CoQ_10_H_2_ before differentiation inhibited adipocyte differentiation and the accumulation of fat. Also, the addition of CoQ_10_H_2_ to differentiated adipocytes reduced the cellular lipid content and promoted lipolysis on Day 5 and Day 10, respectively, after initiating treatment. Taken together, our results demonstrate that CoQ_10_H_2_ improves lipid metabolism and inhibits obesity in KKAy mice.

### CoQ_10_H_2_ regulates liver lipid metabolism

**Figure 3:**
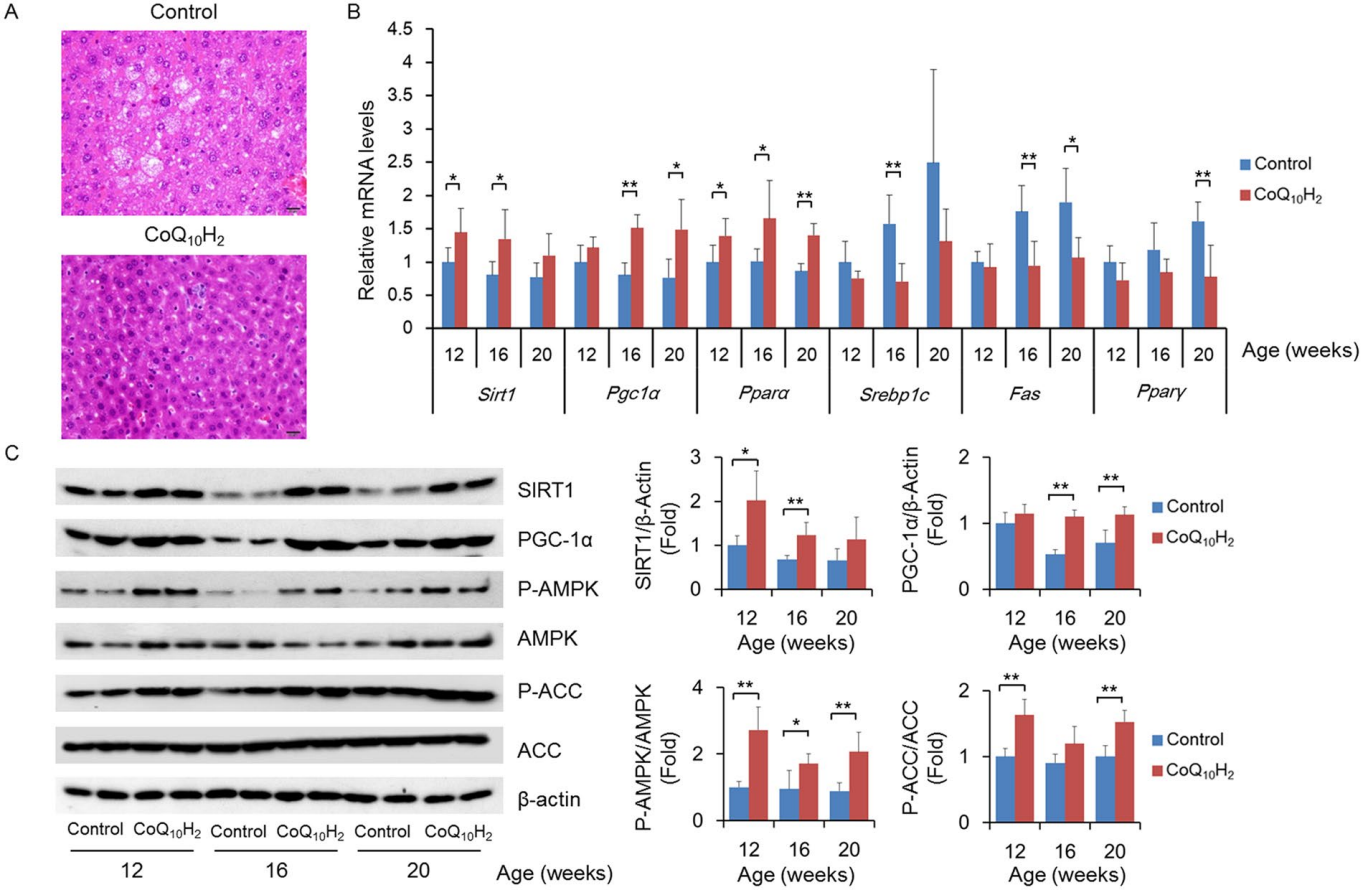
CoQ_10_H_2_ improves cAMP levels and promotes lipid metabolism in the livers of KKAy mice. (**A**) Images of liver tissue stained with H&E in 20-week-old KKAy mice. Bar = 50 μm; (**B**) Changes in gene expressions for factors involved in triglyceride and cholesterol biosynthesis or lipid mobilization and mitochondrial function in 12-, 16-, and 20- week-old KKAy mice in the control and CoQ_10_H_2_ groups as determined by real-time PCR. Histograms show fold-change in mRNA level relative to 12-week-old control KKAy mice. (n = 4–6, ^∗^p < 0.05, ^∗∗^p < 0.01 mean ± SD, Student’s t-test); (**C**) Protein content of SIRT1 and PGC-1α and phosphorylation levels of AMPK and ACC in the livers of KKAy mice were measured by Western blotting. All blots were obtained under the same experimental conditions, and cropped images of the blots are shown. Densitometric quantification is depicted on the right panel. (n = 4–6, ^∗^p < 0.05, ^∗∗^p < 0.01; mean ± SD, Student’s t-test).

The liver plays an important role in the digestion, absorption, synthesis, and decomposition of lipids^46^. We observed H&E-stained liver slices and found that CoQ_10_H_2_ treatment eliminated excessive accumulation of fat in liver cells in KKAy mice (Fig. 3A).

In addition, CoQ_10_H_2_ treatment increased expression of *Sirt1*, *Pgc-1α* and *Pparα*, enhancing mitochondrial function and promoting the β-oxidation of fatty acids, and simultaneously decreased expression of *Serbp1c*, *Fas* and *Pparγ*, thereby inhibiting *de novo* synthesis of fatty acids (Fig. 3B and C). AMP-activated protein kinase (AMPK) acts as an energy sensor to help maintain cellular energy homeostasis; AMPK activation inhibits the accumulation of liver lipids in type 2 diabetic mice and has beneficial effects against hyperlipidemia and atherosclerosis^47, 48^. We assessed hepatic AMPK activity in KKAy mice by measuring the phosphorylation status of AMPK and Acetyl-CoA carboxylase (ACC). We found that hepatic AMPK activity was significantly increased in mice receiving CoQ_10_H_2_ supplementation compared with controls (Fig. 3C).

### CoQ_10_H_2_ inhibits expression of PDE4 in the liver

**Figure 4:**
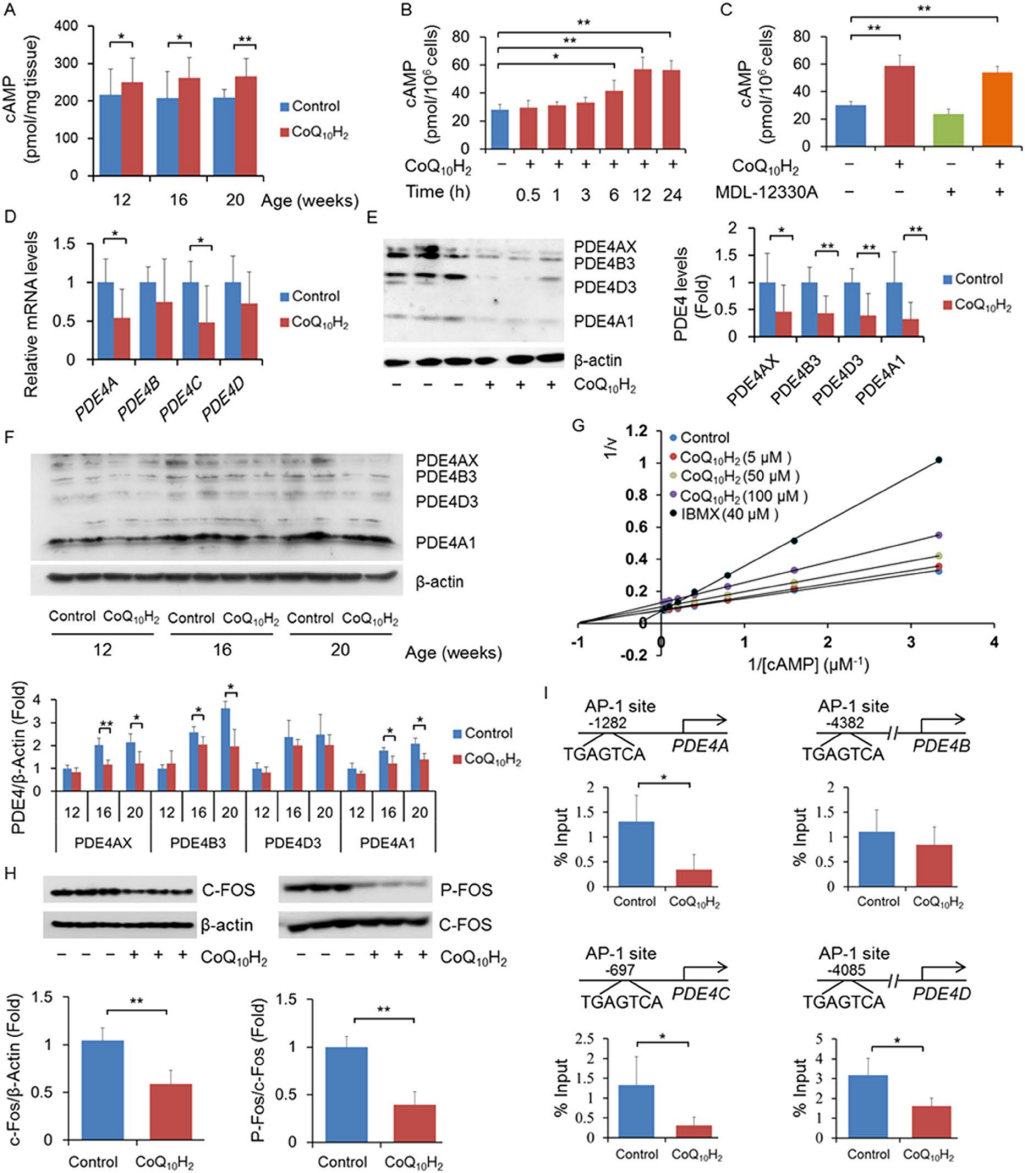
CoQ_10_H_2_ inhibits expression of PDE4 in the liver by decreasing C-FOS binding to the PDE4 promoter. (**A**) Liver cAMP concentrations of KKAy mice in the control and CoQ_10_H_2_ groups at ages 12, 16, and 20 weeks. (n = 4–6, ^∗^p < 0.05, ^∗∗^p < 0.01; mean ± SD, Student’s t-test); (**B**) Change in intracellular cAMP concentration in HepG2 cells 0.5–24 hours after the addition of CoQ_10_H_2_ (5 μM). (n = 6, ^∗∗^p < 0.01; mean ± SD, one-way ANOVA followed by Tukey’s test); (**C**)MDL-12330A (50 μM) pre-treated or untreated HepG2 cells were supplemented with CoQ_10_H_2_ (5 μM) for 24 hours and then intracellular cAMP was measured. (n = 6, ^∗∗^p < 0.01; mean ± SD, one-way ANOVA followed by Tukey’s test); (**D**) mRNA expression levels of PDE4 in HepG2 cells 24 hours after addition of CoQ_10_H_2_ (5 μM) were determined by real-time PCR. Histograms show fold-change of the mRNA level relative to untreated control cells. (n = 9, ^∗^p < 0.05, ^∗∗^p < 0.01; mean ± SD, Student’s t-test); (**E**,**F**) PDE4 protein content in HepG2 cells (**E**) and KKAy mouse liver **(F**) was measured by western blotting. Densitometric quantification is depicted in the right panel (n = 4–6, ^∗^p < 0.05, ^∗∗^p < 0.01; mean ± SD, Student’s t-test); (**G**) Lineweaver-Burk plots show kinetic analysis of PDE activity at 0.3 to 40 μM cAMP. (**H**) Levels of C-FOS protein and its phosphorylation in HepG2 cells 24 hours after CoQ_10_H_2_ (5 μM) supplementation were measured by western blotting. All blots were obtained under the same experimental conditions, and cropped images of the blots are shown (n = 9, ^∗∗^p < 0.01; mean ± SD, Student’s t-test); (**I**) The binding capacity of C-FOS and the PDE4 promoter as detected by ChIP (n = 9, ^∗^p < 0.05; mean ± SD, Student’s t-test).

cAMP is a second messenger that regulates the activity of SIRT1 and AMPK and is involved in regulation of intracellular energy metabolism^49, 50^. We found that dietary supplementation with CoQ_10_H_2_ significantly increased hepatic cAMP content in KKAy mice (Fig. 4A). As a second messenger coupled to G-protein pathways, cAMP concentrations can change rapidly. As such, we measured the change in cAMP content in HepG2 cells 0.5-24 hours after addition of exogenous CoQ_10_H_2_. At 6 hours after the addition of CoQ_10_H_2_, the intracellular cAMP content did not increase significantly, but thereafter cAMP levels displayed a slight upward trend. The cAMP concentration peaked 12 hours after addition of CoQ_10_H_2_ and remained at a high level (Fig. 4B). Adenylyl cyclase (AC) is responsible for intracellular cAMP synthesis and MDL-12330A is a specific inhibitor of AC. Addition of exogenous CoQ_10_H_2_ to cultured HepG2 cells increased intracellular cAMP but was not inhibited by MDL-12330A (Fig. 4C). These results showed that the increased intracellular cAMP induced by CoQ_10_H_2_ is not due to increased cAMP synthesis.

Phosphodiesterases (PDEs) are encoded by 21 genes that are divided into 11 families according to their structural similarity. PDEs regulate cellular signal transduction by specifically hydrolyzing cAMP (e.g., PDE3, 4, 7 and 8) and cGMP (e.g. PDE1, 5, 6, 9 and 10)^51^. In addition, gene expression of PDEs has significant tissue specificity (e.g., PDE3A is mainly expressed in the heart and vascular smooth muscle). Measurement of the expression of each cAMP-specific PDE subtype showed that *PDE4A* and *PDE4C* expression was reduced in HepG2 cells with CoQ_10_H_2_ addition (Figs S2 and 4D). We also confirmed that CoQ_10_H_2_ reduced the protein content of PDE4 in HepG2 cells and the liver of KKAy mice (Fig. 4E and F). To demonstrate whether CoQ_10_H_2_ can directly inhibit PDE activity, we constructed Lineweaver-Burk plots to express the relationship between PDE activity and CoQ_10_H_2_ treatment. The V_max_ and K_m_ of PDEs were not significantly altered- even in the presence of a very high concentration of CoQ_10_H_2_ (100 μM)- compared to IBMX, a nonselective competitive PDE inhibitor (Fig. 4G). These data indicate that CoQ_10_H_2_ enhanced intracellular cAMP concentrations by inhibiting PDE4 gene expression rather than activity.

Activating protein-1 (AP-1), a mammalian transcription factor, is not a single protein, but a homologous or heterologous complex consisting of proteins from the Fos and Jun families^52^. In this study, mRNA levels of the AP-1 component *C-FOS* (but not *C-JUN* (Fig. S3)) and C-FOS phosphorylation were significantly reduced by CoQ_10_H_2_ (Fig. 4H). Next, we investigated the regulatory effect of AP-1 on the expression of PDE4 using chromatin immunoprecipitation (Fig. 4I), and found that AP-1 directly combined with the PDE4 promoter, and that CoQ_10_H_2_ can inhibit the binding of AP-1 to PDE4. These data suggest that CoQ_10_H_2_ regulates binding of AP-1 to the PDE4 promoter, thereby reducing PDE4 levels and inhibiting cAMP hydrolysis.

### CoQ_10_H_2_ regulates calcium signaling pathways by promoting expression and function of SERCA2

**Figure 5:**
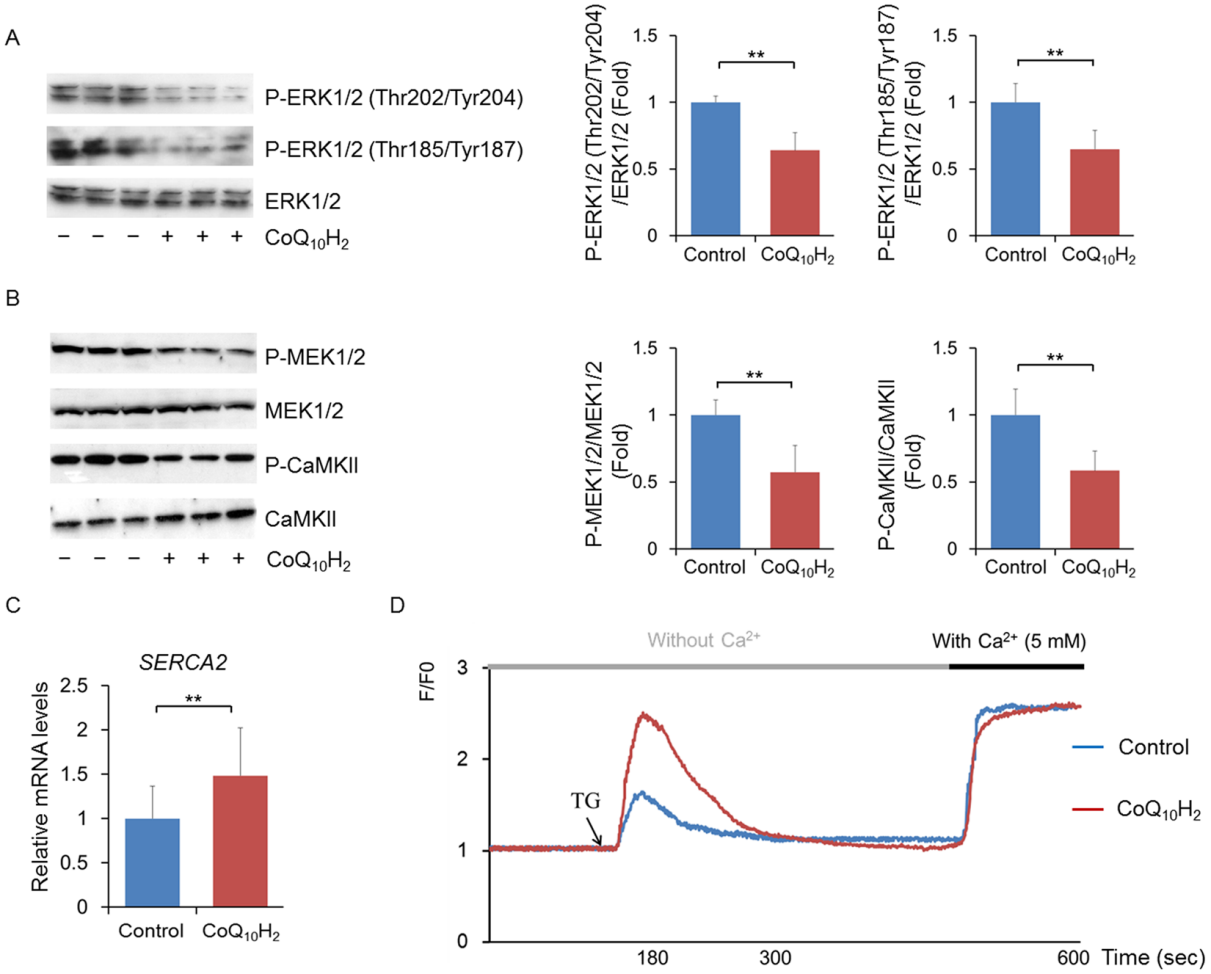
CoQ_10_H_2_ inhibited the activity of CaMKII and MEK1/2 and therefore inhibited phosphorylation of ERK1/2 by enhancing the expression and function of SERCA2. (**A**) Phosphorylated ERK1/2 and total ERK1/2 in HepG2 cells were measured by Western blotting 24 hours after CoQ_10_H_2_ (5 μM) supplementation. All blots were obtained under the same experimental conditions, and cropped images of the blots are shown. Densitometric quantification is depicted in the right panel. (n = 9, ^∗∗^p < 0.01; mean ± SD, Student’s t-test); (**B**) Phosphorylation levels of CaMKII and MEK1/2, the upstream regulators of ERK1/2, were detected by Western blotting in HepG2 cells 24 hours after supplementation with CoQ_10_H_2_ (5 μM). All blots were obtained under the same experimental conditions, and cropped images of the blots are shown. Densitometric quantification is depicted in the right panel. (n = 9, ^∗∗^p < 0.01; mean ± SD, Student’s t-test); (**C**) Gene expression of SERCA2 in HepG2 cells was determined by real-time PCR 24 hours after CoQ_10_H_2_ (5 μM) supplementation. (n = 9, ^∗∗^p < 0.01; mean ± SD, Student’s t-test); (**D**) Determination of intracellular calcium concentration by Flou-3AM (40 μM). HepG2 cells were supplemented with CoQ_10_H_2_ (5 μM) for 24 hours, and then the SERCA2 inhibitor Thapsigargin (Tg) (200 nM) and Ca^2+^ (5 mM free extracellular Ca^2+^ final concentration) were added to the cells, and changes in fluorescence were observed (see the Supplementary Data for details).

As the most classical pathway in the Mitogen-activated protein kinase (MAPK) system, the extracellular signal-regulated kinase 1 and 2 (ERK1/2) pathway transports extracellular signals into the nucleus to activate various effector molecules and regulate biological activities such as cell proliferation and apoptosis via a series of cascade transduction systems^53^. Activation of ERK1/2 promotes the phosphorylation of many transcription factors, including ELK-1, C-FOS and C-MYC, to regulate nuclear translocation and transcriptional activity^54^. ERK1/2 inhibitors, however, inhibit the expression of *C-FOS* in a variety of tissues^55^. For these reasons, we measured phosphorylation of two phosphorylation sites in ERK1/2 in HepG2 cells and found that CoQ_10_H_2_ inhibited phosphorylation of ERK1/2 in cells, while total ERK1/2 content was not changed (Fig. 5A). It is generally believed that the ERK-activated signal cascade requires sequential activation of Ras, Raf1, MAPK/ERK kinase 1 and 2 (MEK1/2), and phosphorylation at Thr185/Tyr187. In addition, CaMKII binds to MEK1/2 and ERK1/2 to form macromolecular complexes to synergistically promote the phosphorylation of ERK1/2 at Thr202/Tyr204 and nuclear translocation^56^. Intracellular phosphorylation of CaMKII and MEK1/2 was inhibited by the addition of CoQ_10_H_2_ (Fig. 5B). Changes in CaMKII and MEK1/2 activity were closely related to cytoplasmic Ca^2+^ concentration. SERCA2 is an important regulator of cytoplasmic Ca^2+^ concentration and works by regulating Ca^2+^ uptake into the ER. CoQ_10_H_2_ increased *SERCA2* expression in both HepG2 cells and KKAy mice liver tissue (Figs 5C and S3), enhanced ER calcium storage, and decreased cytoplasmic Ca^2+^ content (Figs 5D and S1). Our results demonstrate that CoQ_10_H_2_ regulates the activity of factors involved in the Ca^2+^ signaling pathway to inhibit transcriptional activity of ERK1/2 by increasing the expression of *SERCA2*.

## Discussion

It is well known that CoQ_10_H_2_ is a powerful antioxidant that can potently inhibit the generation of oxygen free radicals and oxidative stress damage, thereby ameliorating age-associated disease. This effect has been shown both in mouse and cell experiments^42^. Obesity is a chronic metabolic disease caused by various factors including heredity, environment, dietary patterns, and living habits, and the development of obesity is accompanied by decreased CoQ_10_H_2_ content in adipose tissue, as seen in both human and mouse models^43, 57^. At the same time, increased CoQ_10_H_2_ synthesis can ameliorate metabolic disorders and insulin resistance caused by obesity, and significantly inhibits 3T3L1 preadipocyte differentiation and lipid accumulation^44^. Therefore, we hypothesized that CoQ_10_H_2_ might regulate lipid metabolism to some extent. As a widely used model of obesity and type 2 diabetes, KKAy mice develop obesity and insulin resistance accompanied by hepatic steatosis at an early age^58^. In our study, the development of obesity in KKAy mice was inhibited by administration of exogenous CoQ_10_H_2_, while food intake was not changed (Fig. 1A). At the same time, accumulation of visceral fat was inhibited (Fig. 1B and C) and blood cholesterol and triglyceride content were reduced by CoQ_10_H_2_ treatment (Fig. 1D and E). As a model of insulin resistance, fasting blood glucose in KKAy mice is high and is gradually increased with the aggravation of obesity. These phenomena are potently reversed by CoQ_10_H_2_ supplementation (Fig. 1F–H). We observed the effect of CoQ_10_H_2_ on adipose tissue by H&E staining of adipose tissue. Compared with the control group, adipocyte size in iWAT of KKAy mice was decreased by 42%, and the percentage of lipid droplets in BAT was decreased by 26% with CoQ_10_H_2_ supplementation (Fig. 2A). Analysis of various lipid metabolism markers in iWAT and BAT revealed that CoQ_10_H_2_ can inhibit *de novo* synthesis of fatty acids and promote oxidation of fatty acids (Fig. 2B and C). To determine if CoQ_10_H_2_ affects adipogenesis and lipolysis, we added CoQ_10_H_2_ to pre-adipocyte 3T3L1 cultures before and after differentiation into mature adipocytes. Our results demonstrate that CoQ_10_H_2_ reduces adipose differentiation and lipid storage in adipocytes.

The liver is the main site of lipid metabolism and lipid metabolism disorders caused by obesity are a major cause of non-alcoholic fatty liver disease. Significant hepatic steatosis occurred in the livers of KKAy mice in the control group, while the livers of mice supplemented with CoQ_10_H_2_ did not show obvious changes (Fig. 3A). PGC-1α is a transcriptional coactivator that is closely related to energy metabolism and plays an important role in the process of mitochondrial synthesis and adaptive thermogenesis^59, 60^. PGC-1α is also involved in glucose and lipid metabolism, and has become a new target for the treatment of diabetes, obesity, and other metabolic diseases^20^. Supplementation with CoQ_10_H_2_ increased PGC-1α gene expression in the liver (Fig. 3B). Previous results have shown that expression and activity of PGC-1α are regulated by AMPK and SIRT1^42^. AMPK is an essential protein kinase involved in the regulation of energy metabolism *in vivo*. Activation of AMPK can inhibit ATP-consuming pathways, such as the synthesis of fat and cholesterol, and promote ATP-forming pathways, such as β-oxidation of fatty acids^61^. In a model system, decreased AMPK activity resulted in insulin resistance and activation of AMPK-enhanced insulin sensitivity^62–64^. In addition, AMPK activation was also directly involved in regulating the activity of fat metabolism-related factors, such as SREBP1c^48^. SIRT1 is widely known for its anti-aging effect^65^. SIRT1 also plays an important role in cellular energy metabolism^66^. It directly regulates the expression and activity of PPAR family genes and participates in the regulation of lipid metabolism pathways^67^. In addition, SIRT1 can also deacetylate the AMPK upstream kinase LBK1 and activate AMPK^42^. In KKAy mice, SIRT1 protein content and AMPK activity decrease with age, and this effect is ameliorated by CoQ_10_H_2_ supplementation (Fig. 3C).

The second messenger cAMP plays a key role in transduction of various extracellular signals in cells. Our previous work demonstrated that CoQ_10_H_2_ increases cAMP content in the liver and regulates lipid metabolism in mice^42^. These results were confirmed in the present study (Fig. 4A). We found that specific inhibition of PDE4 expression in cultured cells increased cAMP (Fig. 4D). Interestingly, as shown in Fig. 4G, the specific activity of PDE4 was not changed, indicating that CoQ_10_H_2_ and resveratrol, a known cAMP-SIRT1 activator that acts by directly inhibiting activity of PDE4, do not share the same mechanism of action^50^. We further demonstrated that CoQ_10_H_2_ inhibited transcriptional activity of the transcription factor AP-1 and that of C-FOS, a component of AP-1, and that CoQ_10_H_2_ inhibited the ability of AP-1 to bind to the PDE4 gene promoter (Fig. 4I). By measuring changes in signaling factors upstream of C-FOS, we confirmed that CoQ_10_H_2_ inhibits the transcriptional activity of C-FOS by inhibiting the phosphorylation of ERK1/2. CaMKII and MEK1/2 are both upstream regulatory factors for ERK1/2, and CaMKII and MEK1/2 activity is influenced by changes in cytoplasmic Ca^2+^ concentration (Fig. 5A and B).

Ca^2+^ is one of the most abundant ions in cells. Changes in intracellular Ca^2+^ concentration are a key factor in the maintenance of organelle function and resistance to stress in many metabolic tissues, such as liver and adipose tissue^68^. Regulation of Ca^2+^ levels in the cytoplasm and organelles requires synergistic effects of various signal transduction mechanisms, including the ability to pump Ca^2+^ from the cytosol to the extracellular space or into intracellular reservoirs, such as the ER and mitochondria. Transportation of Ca^2+^ from the cytoplasm into the ER against a concentration gradient requires SERCA2 and energy released from the hydrolysis of ATP. The development of many metabolic diseases, especially obesity and diabetes, is often accompanied by damage to SERCA2, which increases cytoplasmic Ca^2+^ levels, leading to enhanced insulin resistance^9, 18^. CoQ_10_H_2_ inhibits the metabolic disease-induced increase in cytoplasmic Ca^2+^ concentration, but the mechanism is unclear^69, 70^. Our results demonstrate that CoQ_10_H_2_ promotes the expression of SERCA2 and increases ER Ca^2+^ in cultured cells, but has no effect on calcium channels (Fig. 5C and D). Similarly, dietary supplementation with CoQ_10_H_2_ inhibited SERCA2 damage induced by obesity in KKAy mice (Fig. S4).

**Figure 6:**
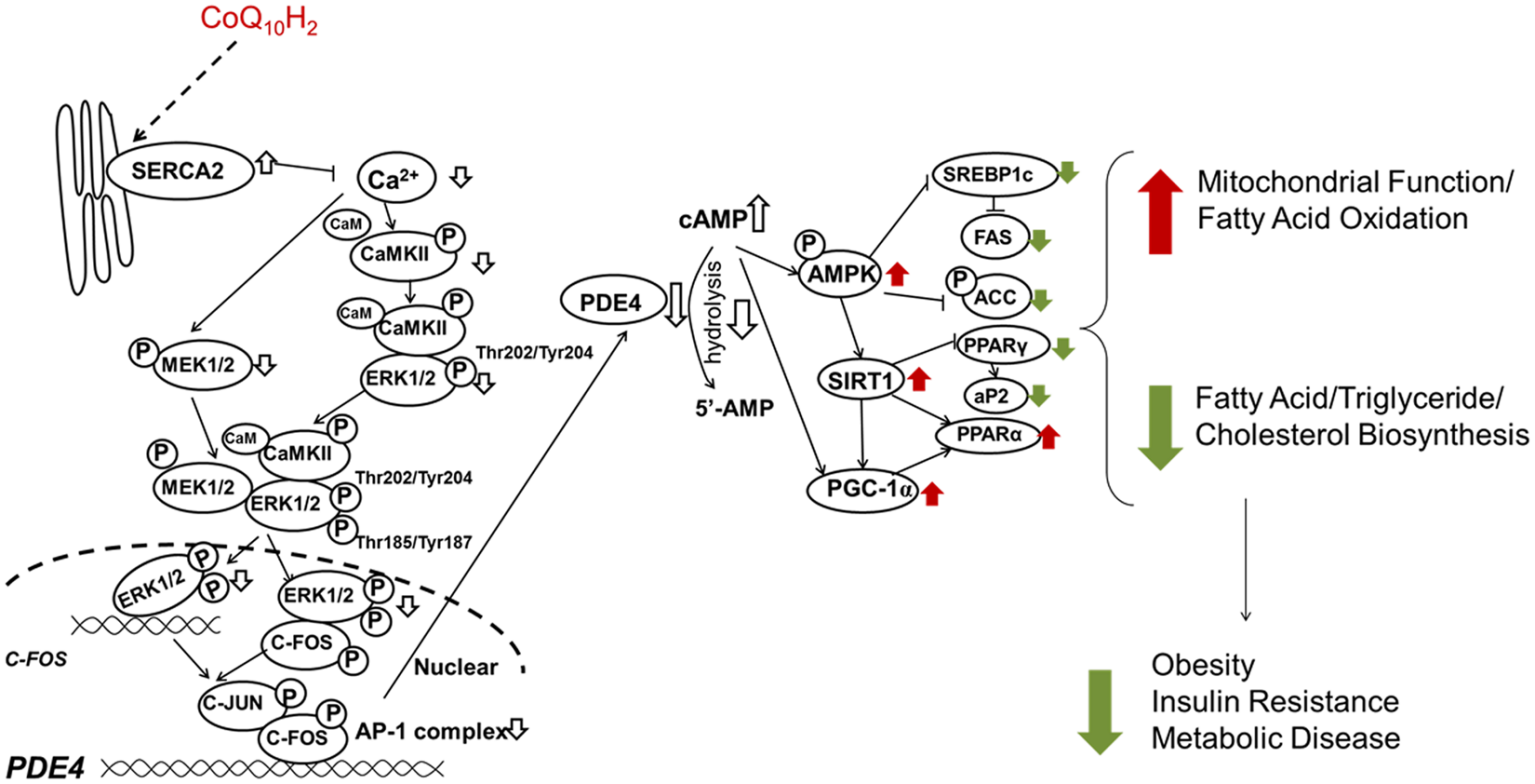
Proposed mechanism by which CoQ_10_H_2_ improves metabolic function and inhibits insulin resistance in KKAy mice. In the liver, CoQ_10_H_2_ inhibited phosphorylation of CaMKII by enhancing the function of SERCA2, and reduced cytoplasmic Ca^2+^, thereby inhibiting the transcriptional activity of the transcription factor C-FOS by regulating the CaMKII-MEK1/2-ERK1/2 signaling pathway. PDE4 gene expression was then inhibited and intracellular cAMP was increased. The increase in cAMP, however, promoted AMPK activity. On the one hand, expression of SIRT1 and PGC-1α was increased and mitochondrial function was enhanced to promote the decomposition of fatty acids. On the other hand, triglyceride and cholesterol biosynthesis was inhibited. In adipose tissue, CoQ_10_H_2_ can prevent the excessive accumulation of visceral fat and promote the function of BAT, thereby improving obesity, insulin resistance, and metabolic syndrome.

Our results demonstrate that CoQ_10_H_2_ promotes the expression of SERCA2 and reduces cytoplasmic Ca^2+^ in the liver cells (Fig. 5C and D). Alteration of Ca^2+^ concentration inhibited the CaMKII-MEK1/2-ERK1/2 signaling pathway, and consequently the transcriptional activity of AP-1. Furthermore, by inhibiting the ability of AP-1 to bind the PDE4 promoter, expression of PDE4 was inhibited and intracellular cAMP was increased. The *de novo* synthesis of fatty acids was inhibited and β-oxidation of fatty acids was enhanced due to CoQ_10_H_2_-induced alterations in the activity of factors such as AMPK and PGC-1α. CoQ_10_H_2_ supplementation also inhibited the accumulation of fat in adipose tissue, promoted fat mobilization, and reduced body weight (Fig. 6). In conclusion, our results suggest that CoQ_10_H_2_ could serve as a safe and effective supplement to improve lipid metabolism and insulin resistance in the future.

## Methods

### Animals

7-week-old female KKAy mice were purchased from CLEA Japan Inc. (Tokyo, Japan) and raised in the Division of Laboratory Animal Research, Research Center for Support of Advanced Science, Shinshu University, under specific pathogen-free (SPF) conditions at 24 ± 2 °C with a light-controlled regimen (12 hours light/dark cycle). The mice were randomly distributed into two experimental groups. Body weight and serum triglyceride and total cholesterol levels were determined and no statistically significant differences were found between the two groups at baseline (data not shown). Animals were fed either CoQ_10_H_2_-supplemented feed (final concentration of 0.3%, mixed with CE-2 standard mouse feed) or CE-2 feed starting from the age of 8 weeks; both diets were purchased from CLEA Japan. All mice were allowed free access to food and water, and body weight was recorded and food intake calculated twice a week. Mice were examined daily. Mice in both groups were fasted for 12 hours at the ages of 12, 16, and 20 weeks, and were anesthetized with sevoflurane (Wako, Osaka, Japan), followed by blood and tissue collection at the end of the experiment.

For the IPGTT, mice were fasted for 12 hours and then given an intraperitoneal injection of glucose (1 g/kg body weight) 3 days before autopsy. Blood samples were collected at different time intervals after injection (0–180 minutes) for blood glucose measurement. Blood glucose levels were measured using Accu-Chek Aviva glucose monitors (Roche, Indianapolis IN). To determine serum levels of insulin, triglyceride and total cholesterol, blood samples were collected from the heart during dissection and stored in test tubes. Serum insulin was measured by ELISA (Morinaga, Yokohama, Japan), and serum triglyceride and total cholesterol levels were measured using enzymatic kits (Wako, Osaka, Japan), according to the manufacturer’s instructions.

All experiments using animals were performed with the approval of the Committee for Animal Experiments of Shinshu University and approved protocols were strictly followed. Permit number: 260066 (from 2015).

### Cell Culture

The human hepatoma HepG2 cell line was provided by the RIKEN BRC through the National Bio-Resource Project of the MEXT, Japan, and 3T3L1 cells were purchased from the Japanese Cancer Research Resources Bank. HepG2 cells were resuscitated and then cultured in an incubator with 5% CO_2_ at 37 °C in DMEM medium (4.5 g/l glucose) supplemented with 10% fetal bovine serum (FBS) and 0.2% antibiotics, and the medium was changed every 3 days. When HepG2 cells reached 90% confluence, CoQ_10_H_2_ (5 μM) was added and cells were cultured for another 24 hours. Cells were then collected with a scraper. Refer to the supplementary data for the experimental protocol for 3T3L1 cells.

### Western blotting and immunoprecipitation

Tissues and cells were lysed in cell lysis buffer (Cell Signaling Technology, MA) supplemented with protease inhibitors (Sigma Aldrich, MO). Protein samples were sonicated, followed by centrifugation at 15,000 g for 10 minutes. Supernatants were collected, and protein concentrations were determined using the BCA protein Assay Kit (Thermo Fisher Scientific, CO). Proteins were separated by electrophoresis at 20 mA for 4 hours on Tris-Tricine/SDS-12% polyacrylamide gels (SDS-PAGE). After electrophoresis, proteins were transferred to a polyvinylidene difluoride (PVDF) membrane using a semidry western blot apparatus at 150 mA for 1.5 hours. The membrane was then probed with the given antibody in 5% milk in TBS-T for 1 hour at room temperature (antibodies are shown in Supplemental Information). Subsequently, membranes were incubated for 1 hour with horseradish peroxidase (HRP)-conjugated anti-rabbit IgG. Target proteins were detected with the enhanced chemiluminescence (ECL) system and quantified using a densitometric image analyzer with Image-Pro Plus 4.5 software (Media Cybernetics Inc., MD).

For immunoprecipitation, lysate (100 μg of protein) was brought to a total volume of 1 ml with lysis buffer containing 0.5 μg antibody and 20 μl protein A/G PLUS–agarose beads (Santa Cruz Biotechnology, CA). The mixture was rotated in a 1.5 ml microfuge tube at 4 °C for 14 hours. Immune complexes were collected by centrifugation at 16,000 g and washed 4 times with chilled lysis buffer and analysed by SDS–PAGE.

### Real-time RT-PCR

Total RNA was extracted using TRIzol Reagent (Invitrogen, CA), followed by treatment with DNA-Free (Applied Biosystems, CA) to remove contaminating DNA and then subjected to reverse transcription using an Omniscript RT kit (Applied Biosystems, CA) with random primers (Applied Biosystems, CA). Quantitative real-time RT-PCR analysis was carried out using an ABI PRISM 7500 Sequence Detection System (Applied Biosystems, CA) with SYBR Green (Takara Bio, Tokyo, Japan). Primer sequences are listed in Supplementary Table 1.

### Cyclic AMP Measurement

Cyclic AMP levels were determined using a cyclic AMP chemiluminescence kit (Cell Signaling Technology, MA) according to the manufacturer’s instructions.

### PDEs activity

For kinetic analysis of PDEs activity, the PDEs, 3′,5′-nucleotides, and cAMP (Abcam plc, Cambridge, UK), were used at concentrations ranging from 0.3 μM to 40 μM of cAMP and the reaction interval was 30 min according to the instrumental procedure. Blanks for spontaneous cAMP hydrolysis contained the corresponding buffer. The kinetic data were plotted as 1/[cAMP] vs. 1/v observed. Michaelis constant (K_m_) and maximum enzyme activity (V_max_) values were calculated from the X and Y intercepts.

### Chromatin immunoprecipitation (ChIP)

The ChIP assay was performed using anti-C-FOS antibody (Santa Cruz Biotechnology, CA) and the Chromatin Immunoprecipitation Kit (Epigentek, NY) per the manufacturer’s instructions. Enrichment analysis was carried out using real-time PCR with specific primers.

### Statistical analysis

All data are presented as means ± SD. Data were analyzed using Student’s t-test or one-way ANOVA followed by Tukey’s test using SPSS for Windows software (version 13.0; SPSS Inc, Chicago, IL). P < 0.05 was considered to be statistically significant.

## Electronic supplementary material

Supplementary Information
