# Supplementary material for: Coenzyme Q10 Improves Lipid Metabolism and Ameliorates Obesity by Regulating CaMKII-Mediated PDE4 Inhibition
Source: Sci Rep. 2017 Aug 15;7:8253. doi: 10.1038/s41598-017-08899-7 (PMC5557856; doi:10.1038/s41598-017-08899-7)
Supplement: Supplementary file 1 — Supplementary Information [file 41598_2017_8899_MOESM1_ESM.doc]

**Coenzyme Q10 Improves Lipid Metabolism and Ameliorates Obesity by Regulating CaMKII-Mediated PDE4 Inhibition**

**Zhe XU^1^ , Jia HUO^1^, Xin DING^1^, Mu YANG^1^, Lin LI ^1^, Jian DAI^1^, Kazunori HOSOE^2^, Hiroshi KUBO^2^, Masayuki MORI^1,3^, Keiichi HIGUCHI^1,4^, and Jinko SAWASHITA^1,4^**

**Supplemental Information**

**Expanded Materials and Methods**

**3T3L1 pre-adipocytes**

Methods for differentiation and oil red O staining of 3T3L1 pre-adipocytes are described in previous studies ^1^. Briefly, resuscitated 3T3L1 cells were cultured in an incubator with 5% CO_2_ at 37°C in DMEM medium (4.5 g/l glucose) supplemented with 10% FBS and 0.2% antibiotics. After 100% confluency was reached, CoQ_10_H_2_ was added to the 3T3-L1 pre-adipocytes (Day 0) and cultured for 24 hours. Differentiation-inducing medium (DMEM containing 10% FBS and 0.5 mM 3-isobutyl-1-methylxanthine, 1 μM dexamethasone, and 1 μM insulin) was used to induce cell differentiation for 2 days, followed by insulin medium (DMEM containing 10% FBS and 1 μM insulin) for 2 days. The medium was subsequently replaced with fresh culture medium (DMEM with 10% FBS) containing insulin (1 μM) every 2 days until complete differentiation. After complete differentiation (Day 5), cells were cultured in DMEM medium with or without CoQ_10_H_2_ (10 μM) for 5, 10, and 15 days, and then lipid content was determined by oil red O staining ^2^.

**Estimation of Ca^2+^ uptake and Ca^2+^ leak of the ER**

ER Ca^2+^ content was measured in several ways, as shown in S1. To evaluate ER Ca^2+^ reuptake, ATP (4 mM) was added to cells for 120 seconds 24 hours after supplementation with CoQ_10_H_2_ (5 μM) to activate SERCA2 and to stimulate Ca^2+^ reuptake into the ER (Supplementary Figure 1A). Then, Ca^2+^-free Hanks buffer containing ethylene glycol bis[-aminoethylether]-N, N, N’,N’-tetraacetic acid (EGTA) (1 mM) was used to rinse off Ca^2+^ and ATP around the ER in order to terminate ER Ca^2+^ uptake (Supplementary Figure 1B). The cells were then treated with fluo-3AM (40 μM) solution and the fluorescence intensity was immediately measured as the baseline (Supplementary Figure 1C). The SERCA2-specific inhibitor Thapsigargin (Tg) (200 nM) was applied 2.5 minutes after the baseline measurement to release all Ca^2+^ from the ER, and fluorescence changes were measured again (Supplementary Figure 1D). When the fluorescence levels stabilized, Ca^2+^ (5 mM free extracellular Ca^2+^ final concentration) was added to induce influx of extracellular Ca^2+^, and the fluorescence was recorded again (Supplementary Figure 1E). Finally, the curve of fluorescence intensity changes from baseline was recorded ^3^.

**Immunoblotting analysis**

The following primary antibodies were used: SIRT1 antibody (Santa Cruz Biotechnology, CA), PGC-1α antibody (Santa Cruz Biotechnology, CA), AMPK antibody　(Cell Signaling Technology, MA), P-AMPK antibody (Thr 172) (Cell Signaling Technology, MA), ACC antibody　(Cell Signaling Technology, MA), P-ACC antibody (Ser 79) (Cell Signaling Technology, MA), PDE4 antibody (Abcam plc, Cambridge, UK), C-FOS antibody (Santa Cruz, Dallas, USA), P-FOS antibody (Ser 362) (Sigma Aldrich, MO), ERK1/2 antibody (Santa Cruz Biotechnology, CA), P-ERK1/2 (Thr 202/Tyr 204) antibody (Santa Cruz Biotechnology, CA), P-ERK1/2 (Thr 185/Tyr 187) antibody (Gene Tex, TX), MEK-1/2 antibody (Cell Signaling Technology, MA), P-MEK-1/2 (Ser 217/221) antibody (Cell Signaling Technology, MA), CaMKII antibody (Santa Cruz Biotechnology, CA), P-CaMKII (Thr 286) antibody (Cell Signaling Technology, MA), β-actin antibody (Bioworld Technology Inc., MN). The secondary antibody used was goat anti–rabbit HRP (Cell Signaling Technology, MA).

**Supplemental Figure Legends**

**Table 1.** Primer sequences

| m*Srebp1c*  GGAGCCATGGATTGCACATT  GGCCCGGGAAGTCACTGT | h*PDE3A*  GATGATAAATACGGATGTCTGTC  ACCGCCTGAGGAGCACTAG |
| --- | --- |
| m*Fas*  GCTGCGGAAACTTCAGGAAAT  AGAGACGTGTCACTCCTGGACT | h*PDE3B*  TGCCTTCTTCTTCCTCACCTG  GACCACCACTGCCACACC |
| m*Ap2*  ACACCGAGATTTCCTTCAAACTG  CCATCTAGGGTTATGATGCTCTTC | h*PDE4A*  TCTCCTCCATCCGTACCTTG  TGGCTTGGAGAAAAATGGTC |
| m*Pparγ*  GCATGGTGCCTTCGCTGA  TGGCATCTCTGTGTCAACCATG | h*PDE4B*  TGATGCTCAGGACATTCTCG  AGTGGTGGTGAGGGACTTTG |
| m*Cd36*  CGCTTTCTGCGTATCGTCGT  GATGCACGGGATCGTGTCT | h*PDE4C*  GACTTACCCCTCGACAACCA  GAAAGTCTGCCTGCCAAGAG |
| m*C/ebpα*  CAAGAACAGCAACGAGTACCG  GTCACTGGTCAACTCCAGCAC | h*PDE4D*  TGCCATCTGTTGATCAGGAA  CCCAGTTGTGTTTCCGAGTT |
| m*Ucp1*  GGCCTCTACGACTCAGTCCA  TAAGCCGGCTGAGATCTTGT | h*PDE7A*  AGATAGGTGCTCTGATACTAG  ATGTCTGTGTCTGGTGTC |
| m*Pgc1α*  TCACCACCGAAATCCTTA  GGTGTCTGTAGTGGCTTGAT | h*PDE7B*  GGCTTCTTGCTCATTTGC  CCTGTTGATGTCTGTTGC |
| m*Cieda*  TGCTCTTCTGTATCGCCCAGT  GCCGTGTTAAGGAATCTGCTG | h*PDE8A*  ATGTTTGCTCGCTTTGGAATC  CAGAATGTGTAGAATTGTGGTAGG |
| m*Dio2*  GTCCGCAAATGACCCCTTT  CCCACCCACTCTCTGACTTTC | h*PDE8B*  CAAATCCCTCCGAGCACAC  CTCCATAAATCTCCTGTTGAAGC |
| m*Elovl3*  TCCGCGTTCTCATGTAGGTCT  GGACCTGATGCAACCCTATGA | h*C-FOS*  TCTGGGTCCTTCTATGCAGCAG  AGCGAGTCAGAGGAAGGCTCAT |
| m*Cox7α1*  CAGCGTCATGGTCAGTCTGT  AGAAAACCGTGTGGCAGAGA | h*C-JUN*  CTCCAAGTGCCGAAAAAGGAAG  CACCTGTTCCCTGAGCATGTTG |
| m*Sirt1*  CCTTGGAGACTGCGATGTTA  GTGTTGGTGGCAACTCTGAT | h*SERCA2*  GGAACCCAAAGGAACCAT  ACAGCCAATAGCCAAGT |
| m*Pparα*  *GCGTACGGCAATGGCTTTAT*  GAACGGCTTCTTCAGGTTCTT | h*β-ACTIN*  GCTCGTCGTCGACAACGGCTC  CAAACATGATCTGGGTCATCTTCT |
| m*Serca2*  CATGCACCGATGGCATTTCCT  CGCTAAAGTTAGTGTCTGTGCT |  |
| m*β-actin*  GACAGGATGCAGAAGGAGATTACT  TGATCCACATCTGCTGGAAGGT |  |

**
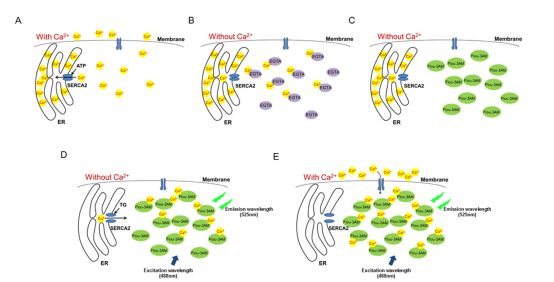
**

**Figure S1** Methodology for measurement of ER Ca^2+^ transport.

For detailed protocol, see the Expanded Materials and Methods section.

**
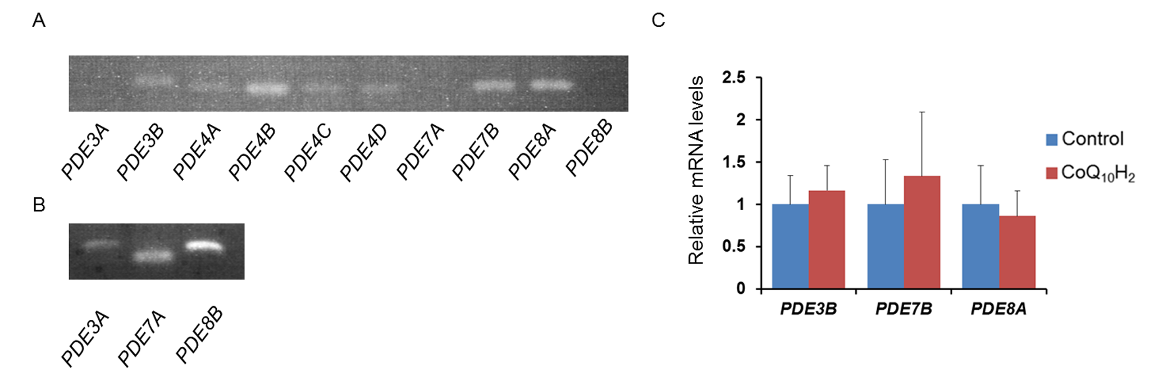
**

**Figure S2** CoQ_10_H_2_ did not alter mRNA expression of PDE3, PDE7 and PDE8.

(A-B) The gene expression of *PDE3*, *PDE4*, *PDE7* and *PDE8* in HepG2 cells was determined by RT-PCR (A); human umbilical vein endothelial cells were used as positive controls (B).

(C) The expression level of *PDE3B*, *PDE7B*, *PDE8A* mRNA in HepG2 cells was measured by real-time PCR 24 hours after addition of CoQ_10_H_2_ (5 μM). (n=8, mean ± SD).


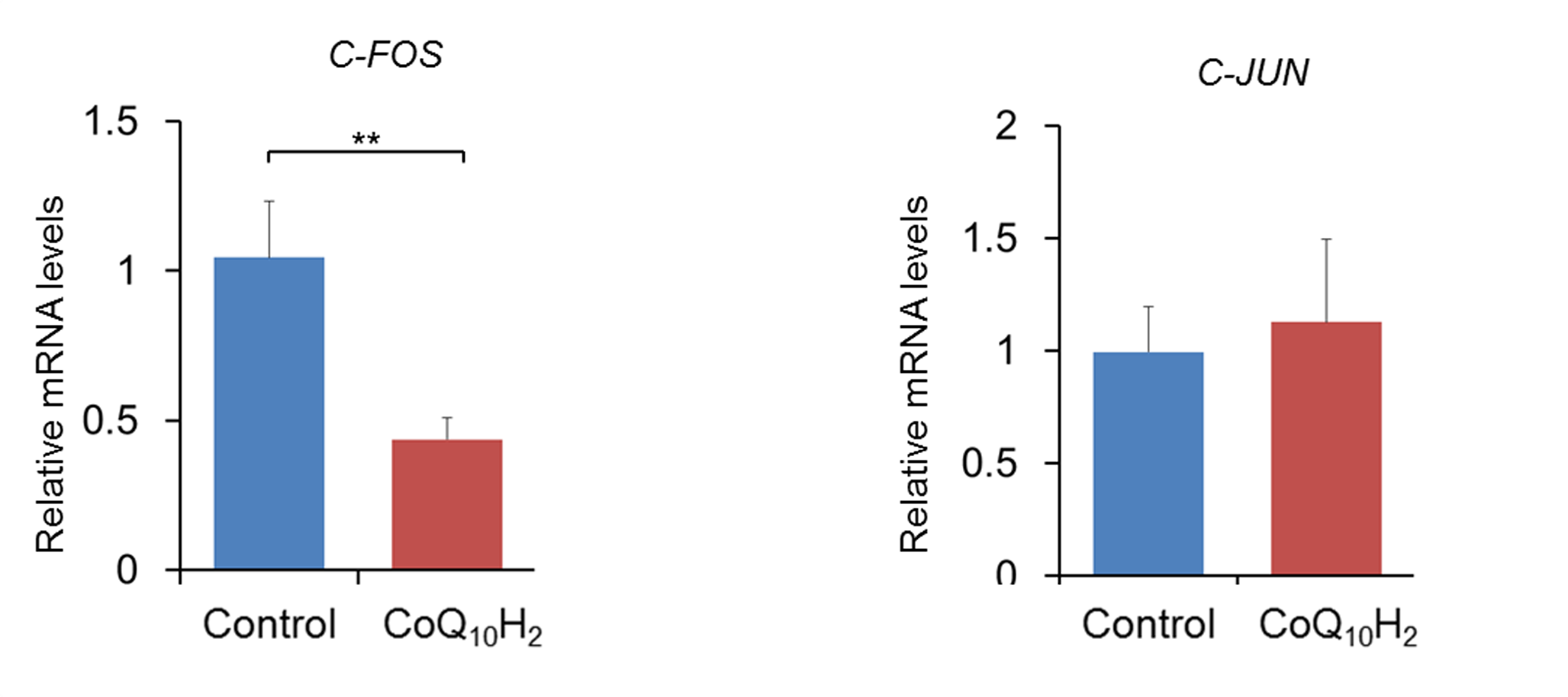


**Figure S3** CoQ_10_H_2_ reduced expression of the AP-1 component c-Fos in HepG2 cells.

Gene expression levels of the AP-1 components c-Fos and c-Jun in HepG2 cells were quantitated by real-time PCR 24 hours after the addition of CoQ_10_H_2_ (5 μM). (n=9, ^∗∗^p < 0.01; mean ± SD).

**
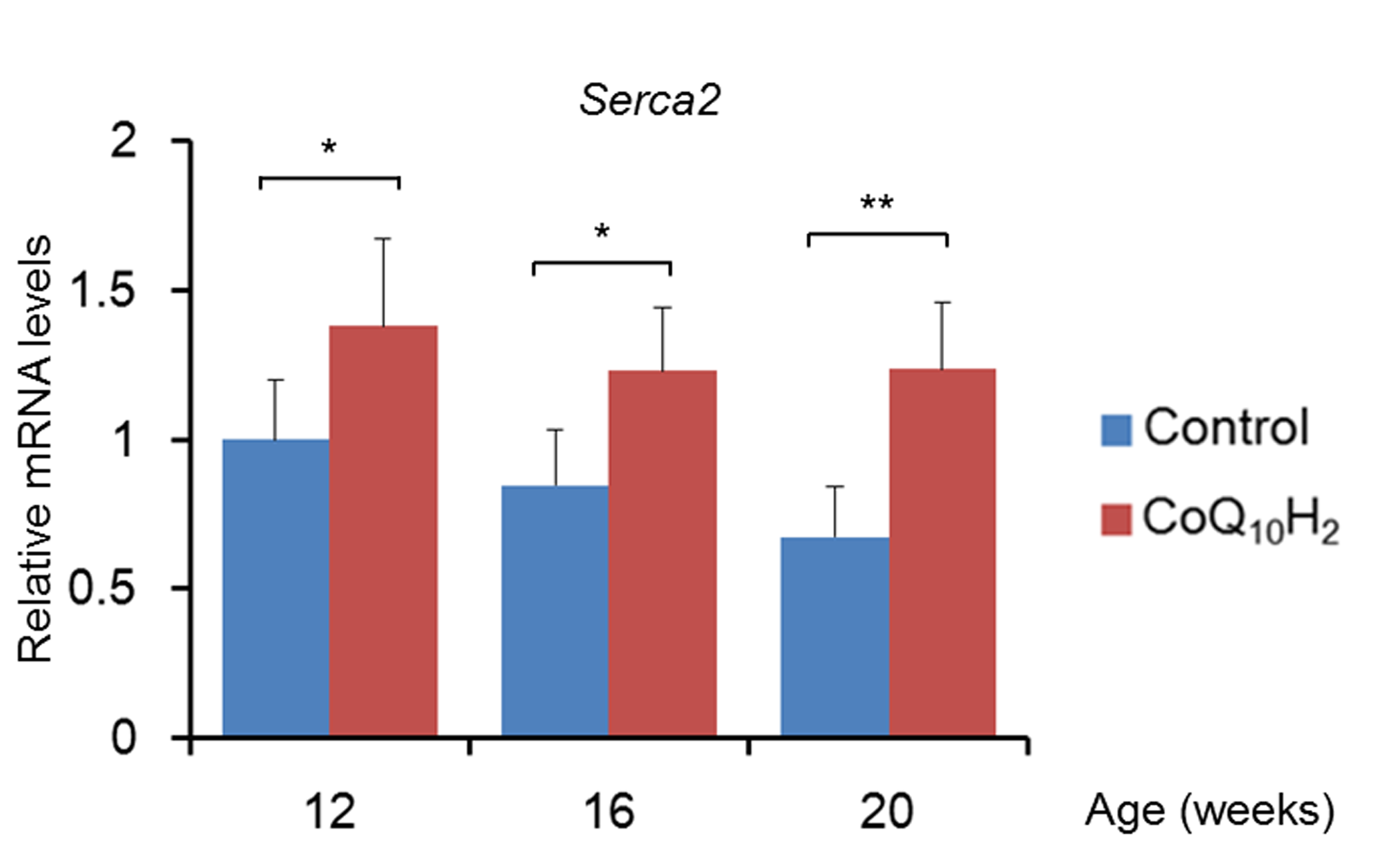
**

**Figure S4** CoQ_10_H_2_ enhanced expression of *Serca2* in livers of KKAy mice.

Changes in gene expression for *Serca2* in 12-, 16-, and 20-week- old KKAy mice in the control and CoQ_10_H_2_ groups were determined by real-time PCR. (n=4–6, ^∗^p < 0.05, ^∗∗^p < 0.01; mean ± SD).

**REFERENCES**

1. Zebisch, K., Voigt, V., Wabitsch, M., and Brandsch, M. (2012). Protocol for effective differentiation of 3T3-L1 cells to adipocytes. Analytical Biochemistry, 425, 88-90.
2. Ramirez-Zacarias, J.L., Castro-Munozledo, F., and Kuri-Harcuch, W. (1992). Quantitation of adipose conversion and triglycerides by staining intracytoplasmic lipids with Oil red O. Histochemistry, 97, 493-497.
3. Scorrano, L., Oakes, S.A., Opferman, J.T., Cheng, E.H., Sorcinelli, M.D., Pozzan, T., and Korsmeyer, S.J. (2003). BAX and BAK regulation of endoplasmic reticulum Ca^2+^: a control point for apoptosis. Science, 300, 135-139.
